# Supplementary material for: iPSC-Cardiomyocyte Models of Brugada Syndrome—Achievements, Challenges and Future Perspectives
Source: Int J Mol Sci. 2021 Mar 10;22(6):2825. doi: 10.3390/ijms22062825 (PMC8001521; doi:10.3390/ijms22062825)
Supplement: Supplementary file 1 [file ijms-22-02825-s001.pdf]

Supplementary material

iPSC-cardiomyocyte models of Brugada syndrome – achievements, challenges and future perspectives

Aleksandra Nijak [1], Johan Saenen [2], Alain J. Labro [3,4], Dorien Schepers [1], Bart L. Loeys [1,5], and Maaike Alaerts [1,*]

[1]Center of Medical Genetics, faculty of Medicine and Health Sciences, University of Antwerp and Antwerp University Hospital, 2650 Antwerp, Belgium

[2]Department of Cardiology, University of Antwerp and Antwerp University Hospital, 2610 Antwerp, Belgium 2

[3]Department of biomedical Sciences, University of Antwerp, 2610 Antwerp, Belgium

[4]Department of Basic and Applied Medical Sciences, Ghent University, 9000 Ghent, Belgium

[5]Department of Human Genetics, Radboud University Medical Centre, 6525 Nijmegen, The Netherlands

*Correspondence: maaike.alaerts@uantwerpen.be; Tel.: +32 032759780

Supplementary Table 1: Overview of the used reprogramming and differentiation approaches, together with described genetic variants, their identification methods and connected clinical phenotypes and patient history. FB- fibroblasts; Sev – Sendai virus; Retro – retroviral vector; Lenti – lentiviral vector; Episomal – episomal vectors; ML – monolayer-based; EB – EB-based; SC – suspension culture; MxS – matrix sandwich); - G – glucose starvation; LT – lactate treatment; Puro αMHC - puromycin selection of αMHC-Puro^r containing spheres; M – male; F – female.

Reference	Reprogramming		Differentiation				Genetic diagnosis			Patient information			
	Cell type	Vector	Method	Agents	Enrichment	Culture period [days]	Gene	Variant	Method of identification	Sex	Diagnosis	Clinical phenotype	Family history
**89**	FB	Sev	ML	Activin A, BMP4, CHIR-90021, Xav939 in BPEL medium	N/A	28-32	SCN5A	c.4912C>T (p.R1638X)	Diagnostic screening	M	BrS	34-year old; BrS type 1 ECG and ventricular tachyarrhythmias during electrophysiological investigations	N/A
								c.468G>A (p.W156X)		M	BrS	52-year old; mild phenotype consisting of first-degree AV block and slight QRS prolongation	
**68**	FB	Sev	ML	CHIR99021, IWR-1	-G	40-60	SCN5A	c.2053G>A (p.R620H) and c.2626G>A (p.R811H)	Diagnostic screening	M	BrS	44 year old; unstable ventricular tachycardia after multiple episodes of recurrent syncope. BrS diagnosis based on BrS type-1 ECG pattern at rest	Sudden death in two paternal uncles and a female cousin
								c.4189delT 4190ΔA/1397Δ(frameshift)		M	BrS	53 year old; recurrent syncope presenting as seizure-like activity. Characteristic BrS ECG changes during procainamide challenge	Sudden death in father and two paternal cousins
**90**	FB	Retro	ML	CHIR99021, IWR-1	LT	35-45	SCN5A	c.677C>T (p.A226V) and c.4885C>T (p.R1629X)	Genetic screening of SCN5A gene	M	BrS	21-year old, presented with syncopal events since the age of 11 during excercise. Baseline ECG with BrS type-2 pattern and first-degree atrioventricular block and QRS duration of 130 ms. Treadmill testing ECG showed a QRS widening uo to 240 ms and a BrS type-1 ECG pattern followed by polymorphic ventricular tachycardia.	Father - p.A226V with ajmaline induced type-1 ECG pattern. Mother - p.R1629X carrier asymptomatic, with baseline ECG showing first-degree AV block at 250 ms, with prolonged QRS duration of 130 ms and a saddle shaped ST elevation in V2 lead. Treadmill challenge revealed QRS prolongation up to 160 ms with no BrS characteristics. Healthy SCN5A variants negative brother and asymptomatic p.A226V carrier sister.
**91**	FB	Episomal	EB	END-2 conditioned media	N/A	22-31	SCN5A	c.1100G>A (p.R367H)	Familial Arrhythmia Network of Scotland	M	BrS	69-year old; classical ST-segment elevation in the ECG	N/A
			ML	CHIR99021, heparin, IWP2									

**56**	FB	Retro	SC<br>ML	CHIR99021, IWP2	N/A	20-46	SCN5A	c.2204C>T (p.A735V)	N/A	N/A	BrS	BrS diagnosis in cases from four different clinical centres	One familial case with multiple mutation carriers with variable phenotypes
**88**	FB	Lenti	EB	END-2 cells co-culture	N/A	50	SCN5A	c.5537insTGA (p.1795insD)	Mutation analysis of SCN5A	M	LQTS3/BrS	Bradycardia, ventricular and atrial conduction slowing, including diagnosed cases of BrS and LQTS	Dutch founder mutation, identified in a large family with multiple individuals presenting variable symptoms
**86**	FB	Retro	EB	Wnt3a	N/A	30	SCN5A	c.5349G>A (p.E1784K)	Genetic screening of SCN5A gene	M	LQTS3/BrS	20-year old; sudden cardiac arrest while driving a car, successfully resuscitated by external defibrillator, which indicated ventricular fibrillation, while surface ECG showed QT interval prolongation. Pilsicainide administration unmasked coved-shaped ST segment elevation.	No family history of previous syncopes or significant QT interval abnormalities
**69**	FB	Lenti<br>Episomal<br>SeV	ML	CHIR99021, IWP2	LT	40-60	SCN10A	c.3749G>A (p.R1250Q) and c.3808G>A (p.R1268Q)	Genetic screening of 8 BrS-associated genes	M	BrS	SCD during ajmaline challenge, no structural abnormalities of the heart	No history of SCD
**70**	FB	Lenti<br>Episomal<br>SeV	ML	CHIR99021, IWP2	LT	40-60	SCN1B	c.629T>C (p.L210P) and c.637C>A (p.P213T)	Genetic screening of 8 BrS candidate genes	M	BrS	Syncope at rest with BrS type 1 ECG in ajmaline challenge	No history of SCD; mutations carrier son with asymptomatic BrS
**71**	FB	SeV	ML (MxS)	tivin A, BMP4, bF	N/A	31-35	RRAD	p.Arg211His	WES	M	BrS	Systematic ECG with typical but labile BrS type 1 ECG pattern with a history of palpitations associated with a near syncope and nocturnal agonal respiration. Treated with an ICD implantation	Identification of 4 affected relatives after flecainide challenge and one with BrS type 1 ECG pattern. No relevant symptoms observed in all of those individuals. History of unexplained SCD in probands' 41-year old uncle. RRAD variant present in all affected individuals (4) and 2 unaffected family members.
**92**	FB	Lenti	EB	Activin A, BMP4, bFGF, DKK1, VERGF, SB-431542	Puro αMHC	60	PKP2	R635Q (c.1904G>A)	Retrospective WES	M	BrS	31-year old; two episodes of syncope at rest with spontaneous BRS type-1 ECG. Implanted ICD with no shocks at follow up	Symptomatic grandfather with sudden death during sleep. Affected mutation-positive father with a history of syncope at rest. Mutation carrier brother with a positive flecainide test

**87**	FB	Lenti STEMCCA	EB	KY02111, XAV939	LT	42-56	Undefined		NGS sequencing of 12 BrS and 26 arrhythmia-related genes	M	BrS	28-years old; VF arrest at 16-years old, spontaneous BrS-type 1 ECG	Father presenting similar ECG
							Undefined			M	BrS	30-years old; ajmaline induced BrS-type 1 ECG, occasional spontaneous	N/A
							PKP2	c.302G>A (p.R101H)		M	BrS	68-years old; ajmaline induced BrS-type 1 ECG leading to VF; occasional spontaneous	N/A
**55**	FB	Lenti STEMCCA	ML	CHIR99021, IWP-4	LT	19	Undefined		Genetic screening of BrS-associated genes	M	BrS	42-year ols; spontaneous BrS type 1 ECG, previous story of syncope, no structural cardiac abnormalities	3 relatives with ajmaline induced BrS ECGs with no history of SCD
							Undefined			M	BrS	67-year old; spontaneous BrS type 1 ECG, previous story of syncope. Diagnosed with BrS during hospital admission for an inferior myocardial infarcion	6 relatives with ajmaline induced BrS ECGs, several family members with previous history of SCD
							CACNA1C	int19 position -7		F	BrS	24-year old; spontaneous BrS type 1 ECG, previous history of syncope, no structural cardiac abnormalities. Inducible non-sustained ventricular tachycardias in RVOT followed by ICD implantation. PR-interval prolongation (220 ms) with normal ORS- and QTc.	5 relatives with ajmaline induced BrS ECGs with no history of SCD

Suplementary Table 2: AP properties published from BrS iPSC-CM models. RMP/MDP – resting membrane potential/maximum diastolic potential; APA – action potential amplitude; APD20/APD50/APD90 – action potential duration at 20/50/90 % of depolarization; V_{max}- upstroke velocity; RT -room temperature.

Reference	Cell line	AP properties										Protocol	In sillico I_{K1} injection
		RMP/ MDP [mV]	APA [mV]	Overshoot [mV]	APD20 [ms]	APD30 [ms]	APD50 [ms]	APD90 [ms]	Vmax [v/s]	peak to peak duration [ms]	n		
**89**	hiPSC-CM^{WT1}	-75±1	105±5				125±20	190±20	56±12		12	Isolated cells recorded on D28-32 of differentiation. APs measured at 37°, elicited at frequency of 1Hz by 3ms	
	hiPSC-CM^{WT2}	-75±0.5	107±3				205±10	290±15	55±3		12		
	hiPSC-CM^{R1638X}	-70±0.5	100±2				160±10	205±15	18±2		14		
	hiPSC-CM^{W156X}	-70±0.5	98±5				150±20	200±20	19±2		16		
**68**	CON1 / CON2	-58.9±0.8	106.4±1.0	48.0±0.7				443.5±17.3	15.6±0.8	49.4±5.5	10to25	APs recorded on dissociated cells at day 40-60 at 36-37 degrees	
	BrS1	-58.1±0.7	107.1±1.6	50.1±1.2				459.3±38.2	11.1±0.1	380.4±96.2	10to25		
	BrS2	-56.5±2.0	105.0±2.6	47.8±2.5				396.2±33.4	7.2±1.1	195.5±60.3	10to25		
	BrSp2-GE	-64.2±1.0	109.3±1.5	46.1±0.9				355.8±19.3	17.4±1.2	48.4±7.4	10to25		
**90**	CON1	-43.39±2.81	87.5±5.76		204.09±44.01		362.09±56.35	473.64±63.97	17.76±4.59	37.83±5.81	11	APs recorded at day 35-45 of differentiation at 34°C	Generated in real time in response to CM membrane potential; potentiometer was set to provide a standard outward current peaked at 150 pA at -75 mV
	BrS_A266V+R1629X	-49.09±6.12	91.16±8.09		277.46±48.5		430.82±60.84	493.18±62	15.76±4.59	32.57±7.92	11		
	BrS_A266V+R1629X 1.0 Hz	-57.83±0.32	73.30±1.53		92.08±5.18		166.10±4.77	221.24±8.58	10.72±0.32		3		
	BrS_A266V+R1629X 1.5 Hz	-57.87±1.54	74.12±0.93		88.61±1.82		153.60±5.87	209.77±9.63	9.21±0.09		3		
	BrS_A266V+R1629X 0.5 Hz	-60.72±2.02	69.00±1.55		95.00±4.82		164.66±9.2	216.36±12.1	14.05±1.87		3		
	BrS_A266V+R1629X 0.2 Hz	-53.52±3.17	64.10±5.35		87.37±7.43		166.30±6.97	221.25±10.31	13.21±0.02		3		
	CON1 with I_{K1} injection	-84.2±04	128.6±1.3	44.4±1.0	126.3±14.2		297.2±25	339.2±26.8	185.1±11		22		
	BrS_A266V+R1629X with I_{K1} injection	-84.4±0.2	115.6±0.9	31.3±0.9	223.8±12.3		318.2±13.9	338±14.3	41.9±5.3		45		

	CON1 with I_{K1} injection 1 Hz	-84.2±0.2	128.0±1.4	43.8±1.1	142.6±18.8		302.9±32.8	348.2±31.7	178.9±12.3		19		
	CON1 with I_{K1} injection 0.5 Hz	-84.3±0.2	129.9±1.3	45.5±1.0	186.0±32.6		381.7±53.0	426.3±52.6	193.8±13.0		19		
	CON1 with I_{K1} injection 0.2 Hz	-84.4±0.2	129.9±1.3	45.5±1.1	133.6±46.5		328.4±65.2	367.2±65.1	197.3±13.9		19		
	BrS_A266V+R1629X with I_{K1} injection non-ER 1 Hz	-84.2±0.4	115.3±0.9	31.10.9	211.7±13.4		310.9±16.1	331.5±16.6	36.9±5.7		33		
	BrS_A266V+R1629X with I_{K1} injection non-ER 0.5 Hz	-84.5±0.4	117.0±1.0	32.7±1.0	308.6±22.8		424.3±26.8	444.2±27.3	40.7±6.4		33		
	BrS_A266V+R1629X with I_{K1} injection non-ER 0.1 Hz	-84.4±0.4	114.1±1.2	29.7±1.2	284.2±29.8		396.0±35.2	414.6±35.8	42.1±6.8		33		
	BrS_A266V+R1629X with I_{K1} injection ER 1 Hz	-85.0±0.3	116.1±2.7	31.1±2.6	173.8±20.4		249.9±17.7	267.4±17.9	36.4±6.9		11		
	BrS_A266V+R1629X with I_{K1} injection ER 0.5 Hz	-84.5±0.4	112.8±2.9	28.3±2.8	148.3±37.0		214.9±39.7	231.8±40.2	36.5±7.4		11		
	BrS_A266V+R1629X with I_{K1} injection ER 0.11 Hz	-81.6±0.9	103.5±4.1	21.9±3.6	31.8±8.7		65.7±10.4	87.6±11.4	35.3±7.0		11		
	CON2 with I_{K1} injection patched at 24°C 1 Hz			40±2				510±100	110±30		9		
	CON2 with I_{K1} injection patched at 24°C 0.1 Hz			42±2				600±190	130±30		9		
	BrS2 p.T1620M with I_{K1} injection patched at 24°C 1 Hz			43±1				370±10	190±20		11		
	BrS2 p.T1620M with I_{K1} injection patched at 24°C 0.1 Hz			44±1				390±20	205±20		11		
	BrS2 p.T1620M with I_{K1} injection patched at 34°C 1 Hz			55±4				505±20	380±10		6		
	BrS2 p.T1620M with I_{K1} injection patched at 34°C 0.1 Hz			56±5				610±90	395±5		6		

	BrS_A266V+R1629X with I_{K1} injection 0.1 Hz	-82.9±0.9	107.6±4.6	24.7±4.0	53.1±21.3		116.2±31.5	127.5±29.7	60.7±12.4		9		
	BrS_A266V+R1629X with I_{K1} injection 0.1 Hz +0.5 mM 4-AP	-83.6±0.6	114.7±1.4	31.1±1.2	405.9±28.18		551.5±23.1	579.2±23.7	52.0±10.0		9		
	BrS_A266V+R1629X with I_{K1} injection 1 Hz	-84.8±0.4	115.6±2.2	30.8±1.9	199.9±18.7		288.9±19.4	308.5±19.7	31.4±7.7		9		
	BrS_A266V+R1629X with I_{K1} injection 1 Hz +Flecainide	-85.0±0.3	109.6±2.1	24.6±1.8	104.0±10.5		157.4±12.3	170.9±12.4	12.6±2.7		9		
**56**	WT (average from both controls)	-70±3	108±4				1802±494		23±7		16	Dissociated cells at 37 degrees patched 20-46 days after the start of differentiation	0 to -100 pA
	WT with I_{K1} injection (average from both controls)	-82±1	135±4				1250±280		121±13		21		
	WT1								140±12		12		
	WT2								98±10		9		
	MUT1								22±4		32		
	MUT2								41±9		29		
	MUT (average from both mutants)	-65±2	104±2				2274±314		7±1		41		
	MUT with I_{K1} injection (average from both mutants)	-82±1	120±2				1350±150		31±5		61		
**88**	hiPSC-CM control	-72.4±0.9	106±3.2		50.7±6.2		89.8±7.9	173.5±12.2	115.7±18.4		16	Single CMs, after day 50 of differentiation; data from 4 independent differentiations per line. Recordings on quiescent cells that contracted upon field stimulation; recordings performed at 36°C; APs elicited at 1,2 or 3 Hz by 3ms, 1.2x threshold current pulses	
	hiPSC-CM het	-71.3±1.3	103.1±3.2		58.7±5.9		109±10.1	217.2±14.9	57.6±14		13		

												through the patch pipette; average from 10 consecutive AP waveforms	
**86**	Control	-65±4	99.3±3.9	34.4±2.5			247.3±30.5	416.7±24	15.8±3.8		10	iPSC-CMs after day 30	
	LQTS/BrS	-60±2.8	97.4±4	37.4±1.8			326.1±74.5	563.7±57.1	21.8±10.8		7		
	corrected	-53.8±1.9	93.1±2.9	39.3±1.2			250.9±40	418±29.6	8.6±1.6		10		
**69**	Control D1	-82±1	138±4				102±13	290±5	42±3		22	Dissociated cells at D40-60 of differentiation, Patching at RT	Yes
	Control D2	-82±1	140±2				142±18	295±5	39±2		21		
	Control D3	-82±1	130±2				98±13	230±5	38±3		22		
	BrS SCN10A	-81±1	125±2				115±15	285±5	23±2		19		
	Control D1 +3 µM ajmaline	-81±1	140±2				90±12	220±5	43±3		11		
	Control D1 +10 µM ajmaline	-81±1	138±2				80±12	219±5	41±3		11		
	Control D1 +30 µM ajmaline	-81±1	135±2				79±12	219±5	36±3		11		
	BrS SCN10A +3 µM ajmaline	-80±1	120±2				116±15	253±7	25±2		15		
	BrS SCN10A +10 µM ajmaline	-81±1	110±2				125±15	280±7	21±2		15		
	BrS SCN10A +30 µM ajmaline	-81±1	108±2				140±15	305±7	20±1		15		
**70**	Control D1	-82±1	138±4				102±13	290±5	42±3		22	Dissociated cells at D40-60 of differentiation, Patching at RT	Yes
	Control D2	-82±1	140±2				142±18	295±5	39±2		19		
	Control D3	-82±1	130±2				100±13	285±5	38±3		30		
	BrS	-82±1	103±2				150±20	320±10	28±3		17		
**71**	Ctl1					150±5	200±10	210±90	14±7	500±100	6	Patched on D31-35 with amphotericin-B on single cells at 37°C; cycle length 700ms	
	BrS1					210±100	290±120	400±200	9±7	1100±1900	16		
	Rad WT					250±60	390±200	400±300	16±29	600±600	7		
	Rad R211H ins					500±110	580±120	590±110	10±3	1100±800	7		
**87**	iPS-HS1M	-58±46	90±35					183.0±17.9	17±10		21	APs recorded at D42-56 of differentiation at 22±2°C	
	iBR1-P5M-L1	-50±10	88±37					125.5±12.4	17±10		17		
**55**	iCtrl 1	-80	115±2		65±6		120±8	155±8	225±13		30		

	iCtrl 2	-80	110±2		85±5		155±8	190±8	200±13		30	iPSC-CMs after day 19 at 36°C; APs elicited at 0.5-3 Hz by 3-ms, ~1.2x threshold current pulses; average from 10 consecutive APs	Constant 2pA/pF
	iBrS 1	-80	118±2		86±5		150±8	175±8	180±20		28		
	iBrS 2	-80	115±2		55±4		100±5	130±5	195±13		30		
	iBrS 3	-78	118±4		80±5		149±8	177±8	260±15		19		
	iSCN5A	-78	126±2		110±5		180±5	240±20	126±25		22		

Supplementary Table 3: I_{Na} properties from published BrS iPSC-CM models. κ – slope factor of activation/inactivation curve; $V_{1/2}$- mid-point of activation/inactivation; τ_f- fast kinetics; τ_s- slow kinetics; I_{NaL}- late/persistent sodium current density; RT – room temperature

Reference	Cell line	Sodium current properties																	Protocol
		I_{Na} peak density		Activation			Inactivation					Recovery			I_{NaL}		Cell capacitance		
		pA/pF	n	κ	$V_{1/2}$ [mV]	n	κ	$V_{1/2}$ [mV]	τ_f [ms]	τ_s [ms]	n	τ_f [ms]	τ_s [ms]	n	pA/pF	n	pF	n	
**89**	hiPSC-CM^{WT1}	-105± 15	11																Recordings at RT; cycle lenght 5s, -5mV hyperpolarizing step from -50 mV
	hiPSC-CM^{WT2}	-102± 13	21																
	hiPSC-CM^{R1638X}	-30± 2	11																
	hiPSC-CM^{W156X}	-32± 3	10																
**68**	CON1	-122.8± 31.3	10-25														22.2± 4	10-25	INa recordings at RT; cycle lenght 40ms; holding potential -80 mV
	CON2																		
	BrS1	-33.7± 6.3	10-25														27± 1.7	10-25	
	BrS2	-36.8± 8.6	10-25														24.9± 2.2	10-25	
	BrSp2-GE	-63.0±4.3	10-25														21.9± 2.6	10-25	
**90**	CON1	-245.8± 32.7	15	4.94± 0.4	-35.72± 0.46	15	7.55± 0.35	-70.26± 0.4			15	19.1± 0.05	327.4± 0.05	14					Patched at RT; cycle lenght 5s; holding potential -90 mV
	BrS_A266V+R1629X	-59.2± 8.8	21	4.99± 0.49	-40.89± 0.56	21	6.38± 0.19	-73.37± 0.22			15	23.3± 0.04	380± 0.05	14					
**91**	Beating bodies Control hiPSC-CM	-78.77± 5.16	3	5.57± 0.33	-44.15± 0.37	3	8.94± 1.54	-61.64± 0.77			3	5.85± 1.01	40.38± 4.95	3					Measurements at RT; cycle lenght 50 ms; holding potential -80 mV
	Beating bodies Patient hiPSC-CM	-42.93± 3.86	7	5.86± 0.28	-36.73± 0.32	7	8.7± 0.57	-70.15± 2.76			7	2.89± 0.35	34.45± 14.45	6					
	Monolayer Control hiPSC-CM	-45.62± 5.37	11	3.94± 0.14	-32.96± 0.79	11	6.7± 0.33	-48.8± 0.79			8	2.58± 0.31	46.17± 7.01	5					
	Monolayer Patient hiPSC-CM	-30.51± 3.09	13	5.58± 0.26	-25.44± 0.78	13	9.59± 0.24	-54.69± 1.21			10	1.68± 0.18	20.12± 6.76	5					

**56**	WT (average from both controls)	-279± 53	20		-58± 0.4	20		-77± 0.3			17	2.4± 0.2	62± 28	17					Measurements at RT; cycle lenght 3s; holding potential -120 mV
	MUT1	-52± 10	31		-31± 2	31		-85± 2			31								
	MUT2	-96± 15	26		-40± 3	26		-84± 2			25								
	MUT (average from both mutants)	-68± 6	57		-35± 0.5	57		-84± 0.1			57	2.7± 0.3	301± 46	57					
**88**	hiPSC-CM control	-264.4± 57	13												0.5± 0.1	9	36± 3.3	13	Measurements at RT; cycle lenght 5s; holding potential -90 mV
	hiPSC-CM het	-121.4± 23.8	13												1.8± 0.2	9	31.7± 3.2	13	
**86**	Control	-200± 10	22												1.8± 0.2	22			Measurements at RT, at rate 0.33 Hz, holding potential of -100 mV
	LQTS/BrS	-180± 20	21												2.5± 0.25	21			
	Control in second experiment with corrected iPSC-CMs	-100± 25	6												0.3± 0.05	6			
	LQTS/BrS in second experiment with corrected iPSC-CMs	-80± 10	13												0.8± 0.1	7			
	corrected	-65± 10	7												0.1± 0.1	13			
**69**	Control D1	-116.9± 28.7	25												-2.6± 0.3 / inhibited -1.1± 0.1	10/ 10			Measurements at RT; cycle lenght 4s; holding potential -100 mV
	Control D2	-95.4 ± 41.6	12												-2.8± 0.4 / inhibited -0.7± 0.2	15/ 24			
	Control D3	-94.7 ± 28.3	11												-3.2± 0.8 / inhibited -1± 0.2	10/ 10			
	BrS SCN10A	-54.7± 9.4	37												-1.1± 0.1 / inhibited -0.6± 0.1	17/ 20			
**70**	Control D1	-116.9± 28.7	25		-53.2± 2.1	25		-73.3± 2.3			25	22± 8		25	-0.4± 0.06	12			Measurements at RT; cycle lenght 4s;

	Control D2	-95.4± 41.6	12		-43.3± 2.1	12		-80.6± 1.5			12	20± 2		12	-0.4± 0.08	16			holding potential -100 mV
	Control D3	-94.7± 28.3	11		-45.1± 4.1	11		-77.9± 2.6			11	22± 2		11	-0.3± 0.006	14			
	BrS	-19.3± 3.7	22		-37.8± 1.8	22		-88.1± 1.8			22	75± 15		22	-0.2± 0.03	14			
**71**	Ctl1	-58.8± 16.5	17	5.3± 0.2	-39.4± 1.3	17	5.3± 0.1	-83.4± 1.3	1.1± 0.1	2.9± 0.3	9	9.7± 0.6	109.1± 6.8	13	0± 1	7	45.7± 22.8	51	Measurements at RT; cycle lenght 50 ms; holding potential -120 mV
	BrS1	-36.8± 16.7	42	5.2± 0.2	-36.1± 0.7	42	4.5± 0.6	-82.9± 0.8	1.2± 0.1	3.5± 0.2	20	6.7± 0.4	93.6± 3.3	33	-3± 5	40	55.5± 25.5	68	
	Rad WT	-56.8± 35.0	18	7.4± 0.1	-34.8± 0.6	9	6.9± 0.1	-83.3± 1.2			9								
	Rad R211H ins	-30.3± 14.2	14	8.4± 0.1	-28.1± 0.4	10	5.7± 0.2	-82.7± 1.5			10								
**92**	H9 hESC-CMs	-100± 60	7																Measurements at RT; 200 ms pusles; holding potential -120 mV
	AC patient iPSC-CMs	-20± 50	8																
	AC patient iPSC-CMs + PKP2 WT	-40± 20	8																
	AC patient iPSC-CMs + PKP2-R635Q	-25± 15	10																
**87**	iPS-HS1M	-110± 18.7	15																INa recordings at RT; cycle lenght 3 s; holding potential -90 mV
	iPS-HS1M +100 µM ajmaline	-15.4± 4.4	15																
**55**	iCtrl 1	-93.6± 84.9	36	6.9± 0.1	-34.6± 0.5	26	6.48± 0.2	-78.9± 1.4			15	1.5± 0.1	5.2± 0.5	36					Measurements at RT; cycle lenght 5s; holding potential -100 mV
	iCtrl 2	-83± 83.3	31	7± 0.2	-33± 0.9	20	7.05± 0.3	-85± 1			11	1.5± 0.1	5.9± 0.7	33					
	iBrS 1	-81.7± 73.25	37	6.6± 0.1	-33.5± 0.5	25	6.6± 0.2	-83.6± 0.9			15	1.52± 0.1	6.4± 0.7	37					
	iBrS 2	-96.8± 70.5	28	7± 0.2	-34.7± 0.7	20	6.6± 0.2	-84.5± 1			10	1.55± 0.1	7.2± 1	31					
	iBrS 3	-151.7± 208.5	31	7.2± 0.2	-32.1± 0.6	29	6.83± 0.2	-80± 0.9			13	1.68± 0.2	7.3± 0.8	32					
	iSCN5A	-38.7± 55.03	25	6.7± 0.2	-31.8± 0.9	20	6.9± 0.2	-83.7± 0.8			10	2.55± 0.2	16.5± 2	25					

Supplementary Table 4: Calcium current (I_{CaL}) properties from published BrS iPSC-CMs. $V_{1/2}$ – mid-point of activation/inactivation; κ – slope factor of activation/inactivation curve; $τ_f$- fast kinetics; $τ_s$- slow kinetics; RT – room temperature.

Reference	Cell line	I_{CaL} properties									Protocol
		Peak		Activation			Inactivation				
		pA/pF	n	$V_{1/2}$ [mV]	κ	n	$τ_f$ [ms]	$τ_s$ [ms]	$V_{1/2}$ [mV]	n	
**56**	WT (average from both controls)	-5.8±0.5	19								Not specified
	MUT (average from both mutants)	-8±1	57								
**69**	Control D1	-9.9 ±1.7	18	-7 ±1.8					-36 ±2.1	18	measurements at RT
	Control D2	-10.1 ±2.8	11	-6.6 ±1.3					-36.2 ±2.2	11	
	Control D3	-6.5 ±1.3	11	-5.5 ±1.5					-34.6 ±1.3	11	
	BrS SCN10A	-3.4 ±1.5	12	12.3 ±4.1					-47.1 ±3.2	12	
**70**	D1	-8.0±1.4	20	-7±2		20		15±5	-35±2	20	Not specified
	D2	-8.4±1.9	18	-6.5±1.5		18		13±6	-35±3	18	
	D3	-6.5±1.3	11	-5,9±1,1		11		11±5	-33±2	11	
	BrS	-7.4±2.4	13	-5±5.2		12		33±8	-33±9	13	
**71**	Ctl1	-26.3±7.1	34	-18.6±0.6	5.5±0.1	34					Not specified
	BrS1	-18.9±6.0	24	-18.6±0.6	5.8±0.1	24					
	Rad WT	-6.1±1.7	6	-14.1±0.6	6.7±0.4	6					
	Rad R211H ins	-1.7±0.5	8	-11.2±0.6	6.2±0.3	8					
**55**	iCtrl 1	-52±3	21				4.0±0.2	18.2±0.8			measurements at 36°C; cycle lengths 2s
	iCtrl 2	-58±3	21				3.7±0.2	21.5±1.3			
	iBrS 2	-60±3	19				3.6±0.3	21.5±1.4			

Supplementary Table 5: Calcium transient properties from published BrS iPSC-CMs. CTD50/75 – calcium transient duration at 50/75% of depolarization.

Reference	Cell line	CT properties						Protocol
		CTD50 [ms]	CTD 75 [ms]	Beat interval [ms]	Rise rate [ms]	Amplitude [mV]	n	
68	CON1	471.3±12.6		108.3±20.4	92.0±6.6	6±0.3	10-25	CT measured on dissociated cells with 5 µM Fluo-4 AM and 0.02% Pluronic F-127 at 37°C.
	CON2							
	BrS1	1362.7±124.6		1894.7±436.5	10.3±0.9	2.6±0.2	10-25	
	BrS2	541.5±60.2		313.3±59.1	45.2±4.7	2.3±0.2	10-25	
	BrSp2-GE	589.3±27.4		190.7±24.6	72.7±5.0	4.7±0.1	10-25	
71	Ctl1		500±150				5	CT measured on single CMs at 37°C.
	BrS1		790±180				7	
	Rad WT		390±400				22	
	Rad R211H ins		590±400				27	

Supplementary Table 6: Field potential properties from published BrS iPSC-CMs. FPD – field potential duration; BPM – beats per minute.

Reference	Cell line	FPD						Protocol
		Baseline [ms]	100 nM ajmaline [ms]	1 µM ajmaline [ms]	10 µM ajmaline [ms]	100 µM ajmaline [ms]	Beating rate [BPM]	
86	Control	210±5					65±17	Not specified
	LQTS/BrS	350±50					59±6	
87	iPS-HS1M	322.3±19.1	326.0±19.3	334.4±19.0	379.8±29.4	462.1±47.4		Sampling frequency 10 kHz at 37°C. One minute baseline recordings were taken after minimum 15 min superfusion of basal media, and 1 min drug recordings were taken after 6 min superfusion.
	iBR1-P5M-L1	319.6±12.8	327.4±13.6	352.1±12.6	385.7±13.3	415.2±26.1		
	iBR1-P5M-L9	281.3±15.0	307.7±13.8	328.7±15.0	364.8±16.4	418.7±23.1		
	iBR1-P3M-N2 (PKP2-R101H)	395.5±10.9	439.3±7.9	448.0±13.9	505.0±21.8	596.6±19.5		
	iBR1-P6M-L1	385.4±32.9	410.1±32.8	428.7±29.7	484.0±30.1	553.0±27.0		

Supplementary Table 7: I_{to} properties from published BrS iPSC-CMs. $V_{1/2}$- mid-point of inactivation

Reference	Cell line	I_{to} properties					Protocol
		Peak		Inactivation			
		pA/pF	n	Time constant [ms]	$V_{1/2}$	n	
**56**	WT (average from both controls)	15±1	18				Not specified
	MUT (average from both mutants)	6±1	59				
**70**	Control D1	2.7±0.4					Not specified
	Control D2	1.9±0.6					
	BrS	1.7±0.4					
**55**	iCtrl 1	14.5±2	13	28.1±4.6		13	Measurements at 36 °C; cycle length 10s
	iCtrl 2	11.5±2	13	26±5		13	
	iBrS 2	14.5±2.5	13	27.1±3.3		13	

Supplementary Table 8: I_{Kr} properties from published BrS iPS-CMs.

Reference	Cell line	Peak I_{Kr}				Protocol
		pA/pF	n	pA/pF +100 µM ajmaline	n	
**70**	Control D1	2.5±0.25	19			Not specified
	Control D2	2.2±0.4	11			
	BrS	1.1±0.2	13			
**87**	iPS-HS1M	1.7±0.2	8	0.7±0.1	8	Not specified

Supplementary Table 9: I_{Ks} properties from published BrS iPSC-CMs.

Reference	Cell line	Peak I_{Ks}		Protocol
		pA/pF	n	
**64**	Control D1	1.2±0.2	21	Not specified
	Control D2	1.25±0.25	14	
	BrS SCN10A	0.3±0.2	7	
**65**	Control D1	0.7±0.2	16	Not specified
	Control D2	0.7±0.3	13	
	Control D3	N/A		
	BrS	0.1±0.09	10	
